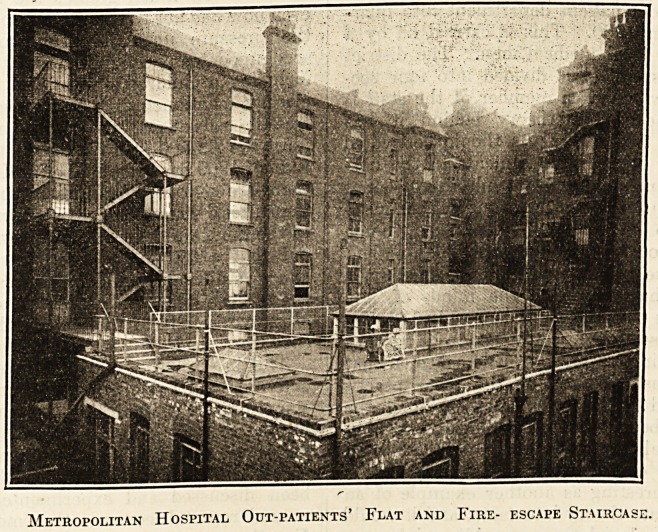# The Progress of the Metropolitan Hospital

**Published:** 1911-09-30

**Authors:** 


### HOSPITALS OF TO-DAY.

### X.—THE PROGRESS OF THE METROPOLITAN HOSPITAL.

With the conference of the British Hospitals' Association this week the Metropolitan Hospital is associated in an intimate manner from the fact that the Secretary and House Governor, Mr. J. Courtney Buchanan, is reading a paper on the "Voluntary Hospitals System and the Insurance Bill." It will, therefore, be of interest to summarise the position and needs of this hospital at the present moment, for this position and these needs may perhaps illuminate certain of his statements, and are certainly worth stating from their intrinsic interest.

A glance at the files of The Hospital reminds us that in November, 1909, the Metropolitan Hospital re-opened its doors after having been closed during four months for thorough reconstruction. In the light of what follows this reconstruction is noteworthy, for experiments in theory, as well as in practice, have marked the development of this institution. Under the chairmanship of Lord Howard de Walden and the Treasurership of Mr. Leopold de Rothschild, the Chairman of Committee, Mr. C. J. Thomas, the Committee itself, Mr. Courtney Buchanan, and the honorary staff have paid a good deal of attention to the theory as well as to the necessary routine practice of their work. The following are some interesting examples.

Some years ago a member of the medical staff, with the approval of the Committee, succeeded in starting a "prevention of disease" department, the object of which was to bring to the homes of the patients some of the lessons of hygiene and simple treatment which they learnt in the wards at times of serious illness, and for this purpose a sister was told off to assist in completing certain of the simpler treatments in the patients' homes. It was an enterprising suggestion and experiment, the result doubtless of that sense of despair from which all intelligent members of a hospital staff suffer at intervals, when the fruitlessness of treating cases at the inevitably late stage at which they appear, and the difficulty of enforcing preventive measures, is borne in upon them. The experiment, though it was discontinued, was not profitless. It taught, among other lessons, the importance of having the home visiting carried on in relation to the hospital, the medical staff of which is personally acquainted with the cases. The cause of its discontinuance was the fear of overlapping the work which the local health visitors attempted to do. As these were already at work, the field was theirs, but, while gracefully leaving it to them, the hospital authorities formed the idea, or cherished the dream, of a future institution which should become a preventive as well as a healing centre from which should radiate lessons in hygiene, science, and treatment for the benefit of students not merely medical, the staff, patients, and district drawing inspiration and knowledge from a corporate life.

Metropolitan Hospital Out-patients' Flat and Fire- escape Staircase.

Since the discontinuance of the "prevention of disease" department a new official has sprung up in hospital life, whom the Metropolitan Hospital was one of the first institutions to adopt. We refer, of course, to the almoner. Few now see in this official an inquisitor whose business it is to detect and expose abuse in the out-patient department. His creation may have owed something to that spirit; but the importance of his office is due to a positive and constructive function. He has to see that the lessons learnt in the wards are not forgotten; that recovery shall be converted into convalescence; that the hospital's duty to the patient does not end with his dismissal; that, in short, he must be assisted to start again with the crutch or the holiday without which he is crippled still. It should be added that the office here is held by a gentleman, and not by a lady, Mr. T. W. Cramp being entrusted with the work. He has had, of course, a special training, having spent six months with the Charity Organisation Society, and is on the Almoners' Board. Through the Samaritan Fund he is brought into close contact with the secretarial department. The Insurance Bill, by the way, adumbrates the visiting by district nurses of certain classes of patient, and if this could be done under the supervision of the doctors who actually treat each case, the Metropolitan Hospital Committee would, we believe, regard it with sincere approval. But such supervision, or, rather, co-ordinated action, again points to the almoner, for he does on a small scale what the great funds do for the hospitals of London—that is to say, he co-ordinates the needs of his district by supervision and assistance. In the future of the almoner's department the authorities of the Metropolitan Hospital have great faith.

Another interesting experiment of this hospital is the Provident Dispensary, founded in 1887, and moved from the out-patient department into quarters of its own in 1900. This is carried on by a paid staff of four local medical men. The district served by the dispensary is divided into four sections, to one of which each member of the staff is peculiarly attached. The theory behind it has always been that trivial ailments should be treated in the dispensary and serious ailments in the hospital. Monthly payments entitle subscribers—that is, poor people who reside within a mile of the institution—either to out-patients' attendance, home visits from the local practitioner, or to in-patient treatment. At the present moment the dispensary's future is somewhat exercising the minds of Mr. Buchanan and his Committee. Clause 14 of the Insurance Bill, sub-section 4, is expected to be perhaps a cause of stumbling. Will not, it is asked, if that clause is passed as it stands, all the local practitioners be affected? If so, the lines on which the dispensary has been run hitherto will probably require a good deal of modification. We cannot go into that on this occasion, but the future of this dispensary is interesting as another example of an attempt to translate theory into facts, and it would be curious if future legislation, or public opinion, as in the previous illustration of the almoner, again promulgated lines of activity that had been tried as experiments in Kingsland Road.

So much for the theory that inspires much of the work of the Metropolitan Hospital. Now for the practice. This, as is often the case, has really been conditioned by the hospital's site, which forms a triangular space of 2½ acres facing Kingsland Road, with its apex out of sight from that quarter. A large portion of the site is at present occupied by houses, but in the future the whole space is likely to be available. Space is wanted now for at least one purpose. The Metropolitan Hospital is without a nurses' home; the nursing staff is housed on the top floor in a series of cubicles which have been adapted for the purpose, for originally the ward-maids were quartered here. The latter have now been moved to quarters of their own in a building contiguous to the out-patient department. There is no need to emphasise this need. It is only to be hoped that the working men of the district, and the City men, on whom the hospital relies jointly for the bulk of its support, will remember how its work is hampered by this state of affairs. Were a nurses' home built, not only would the nursing staff be housed, as they deserve to be housed, away from their work, but thirty-six more beds would be at the disposal of the district. This, of course, would mean the conversion of the present nurses' quarters into two wards, and, administratively, would be a very valuable development. The general cost per patient would be lessened at the very moment when their numbers would be increased.

An important and not too widely known side of the hospital's work is that undertaken for Jewish patients. Two wards are devoted to them, and, of course, a kitchen, where the Koscher food is specially prepared. The out-patient department last year had to cope with 153,000 attendances. It receives patients twice a day, and it is disquieting to think how much greater the number would be even now had not the dispensary been able to lighten the pressure upon it. The hospital at present accommodates one hundred and twenty-three beds, including a children's ward and the Jewish wards already mentioned, but nothing can be done to set free the beds at present occupied as nurses' quarters until the £2,500 still owing on the improvements fund of 1909 has been paid off. It will be seen that there are serious difficulties in the way of building the nurses' home, which is so much wanted. As to the nurses themselves, it is worth while pointing out that a training at the Metropolitan Hospital offers an advantage which is necessarily excluded at those hospitals which possess a medical school. More work falls to their share than would be the case were students attached to the institution.

This brief outline of the Metropolitan Hospital's activities will have served its purpose if it conveys something of the spirit under which the work has been carried on. That, after all, is one of the most important characteristics of the voluntary hospital. Carry away some impression of the ideas that have been discussed and experimented upon, and the annual report becomes rather more like literature. For three and a-quarter years Mr. Buchanan has been secretary, and his extra-hospital work is known through his book, "The Functions of the Voluntary Hospitals in Relation to the Proposed Public Assistance Authority," which was reviewed in THE HOSPITAL of March 4 last (page 663). Previously to his appointment at the Metropolitan Mr. Buchanan had been in charge of the building, furnishing, and equipment of the new Bolingbroke Hospital, and before that had been for nine years at St. Bartholomew's Hospital.

The needs and position of the Metropolitan Hospital take significance from the fact that theory and practice have equally inspired the hospital's Board in its attempt to satisfy them, and on that ground an unhackneyed appeal can be based for further support.

## Figures and Tables

**Figure f1:**